# Plantar Pressure Variability and Asymmetry in Elderly Performing 60-Minute Treadmill Brisk-Walking: Paving the Way towards Fatigue-Induced Instability Assessment Using Wearable In-Shoe Pressure Sensors

**DOI:** 10.3390/s21093217

**Published:** 2021-05-06

**Authors:** Guoxin Zhang, Duo Wai-Chi Wong, Ivy Kwan-Kei Wong, Tony Lin-Wei Chen, Tommy Tung-Ho Hong, Yinghu Peng, Yan Wang, Qitao Tan, Ming Zhang

**Affiliations:** 1 Department of Biomedical Engineering, Faculty of Engineering, The Hong Kong Polytechnic University, Hong Kong 999077, China; guo-xin.zhang@connect.polyu.hk (G.Z.); duo.wong@polyu.edu.hk (D.W.-C.W.); ivy-kk.wong@polyu.edu.hk (I.K.-K.W.); tony.l.chen@connect.polyu.hk (T.L.-W.C.); tommyth.hong@connect.polyu.hk (T.T.-H.H.); 18041923r@connect.polyu.hk (Y.P.); yawang@polyu.edu.hk (Y.W.); matthew.tan@connect.polyu.hk (Q.T.); 2 Shenzhen Research Institute, The Hong Kong Polytechnic University, Shenzhen 518057, China

**Keywords:** prolonged walking, gait parameters, gait instability, pedobarography, balance

## Abstract

Evaluation of potential fatigue for the elderly could minimize their risk of injury and thus encourage them to do more physical exercises. Fatigue-related gait instability was often assessed by the changes of joint kinematics, whilst planar pressure variability and asymmetry parameters may complement and provide better estimation. We hypothesized that fatigue condition (induced by the treadmill brisk-walking task) would lead to instability and could be reflected by the variability and asymmetry of plantar pressure. Fifteen elderly adults participated in the 60-min brisk walking trial on a treadmill without a pause, which could ensure that the fatigue-inducing effect is continuous and participants will not recover halfway. The plantar pressure data were extracted at baseline, the 30th minute, and the 60th minute. The median of contact time, peak pressure, and pressure-time integrals in each plantar region was calculated, in addition to their asymmetry and variability. After 60 min of brisk walking, there were significant increases in peak pressure at the medial and lateral arch regions, and central metatarsal regions, in addition to their impulses (*p* < 0.05). In addition, the variability of plantar pressure at the medial arch was significantly increased (*p* < 0.05), but their asymmetry was decreased. On the other hand, the contact time was significantly increased at all plantar regions (*p* < 0.05). The weakened muscle control and shock absorption upon fatigue could be the reason for the increased peak pressure, impulse, and variability, while the improved symmetry and prolonged plantar contact time could be a compensatory mechanism to restore stability. The outcome of this study can facilitate the development of gait instability or fatigue assessment using wearable in-shoe pressure sensors.

## 1. Introduction

Population aging is a global triumph challenging the existing models of social and healthcare. There are approximately 700 million elderly people, which accounts for about 10% of the world’s population [1]. The elderly population is expected to increase to over 1.5 billion in the next 30 years [1]. Healthy and active aging becomes the top priority to enable wellbeing at older ages and relieve the burden of public health resources. Different research and campaigns have endeavored to maintain and improve physical fitness for elderly people.

Elderly people shall integrate regular physical exercises into daily routines [2,3], such as walking and jogging [4,5]. It was suggested that older adults shall walk 7000 to 10,000 steps a day for health benefits. Walking at least 5000 steps a day can maintain muscular strength and functional mobility for the elderly that could effectively reduce the risk of falling [6]. Unfortunately, the majority of the elderly people could barely reach the goal and could only take approximately 3500 steps a day [7,8]. Fear of falling could be one plausible reason since the elderly are believed to more easily get fatigued and fall [9]. Falling is a significant cause of morbidity and mortality in elderly people [2]. To this end, we believe that recognizing the fatigue state and gait derivation/instability could provide a timely reminder to elderly people, which can minimize the risk of falls. In addition, this could help the elderly overcome fears and encourage them to do more physical exercises; improving their muscle strength, coordination, and balance ability; and thus reducing the risks of falling [10].

The influence of fatigue or instability on the gait parameters from different perspectives has been reported in previous studies, such as spatiotemporal parameters [11], ground reaction force [12], joint angle and angular velocity [13], the center of gravity displacement [14], etc. The variability of temporal measures of stance and swing [15], cadence [16,17], stride velocity [15,18,19], step width [9], and center of mass [17,20] have been proved to be related to lower limb fatigue, instability or fall risk of elder adults. Elderly with fall experiences walked with smaller ankle plantar flexion and hip extension in the dominant leg during push-off in addition to the reduction of peak moment of the knee muscles [17,21,22]. Fatigue was also found to induce the asymmetry of ankle angular velocity, the variability of ankle angular velocity on both sides, and the variability of the knee at the non-dominant side [13]. Minimum foot clearance was another measure to evaluate aging-associated gait instability [23,24], while its asymmetry and variability were regarded as an indicator of fall risks [9,25].

However, these experiments were highly dependent on laboratory-based measurements, such as using the motion capture system and force platform. Nowadays, the development of wearable devices, such as inertial measurement units [26,27], surface electromyography [28], and in-shoe plantar pressure measurement [29,30] enables gait assessments of fatigue in a field setting. In particular, studies using plantar pressure measurement discovered pressure shifts due to local fatigue of toe flexors and an attenuated shock absorption ability explained by weakened muscular control [31,32,33]. The advantage of this study was that the plantar pressure sensors are portable and thus measurement or assessment would not be confined into a laboratory setting with mounted equipment. We believed that the dynamical system theory [34], including the asymmetry [35] and variability parameters [36], may also apply to the plantar pressure measurements to assess instability, and thus fatigue. The objective of this study was to explore the changes of asymmetry and variability of plantar pressure metrics in different plantar foot regions upon prolonged brisk walking on treadmills among the elderly. We presumed that fatigue was induced by the brisk walking and would be verified by their verbal feedback. The hypothesis of this study was that the elderly would experience fatigue after the brisk walking tasks and may have an elevated asymmetry and variability in plantar pressure.

## 2. Materials and Methods

### 2.1. Participants

The sample size of this study was estimated by a priori power analysis using the software, G*Power 3.1.9.7 (Universität Düsseldorf, Düsseldorf, Germany) [37]. The estimation was 15 based on a significance level of 0.05, statistical power of 0.8, and a single factor with 3 repeated measures at a correlation level of 0.5 using an F-test within-factor model. The assumed effect size was medium to large (Cohens f = 0.35) based on a review summary on relevant studies [38]. Eighteen older adults were recruited from the university campus by posters and leaflets. Three of them were excluded due to left foot data errors and re-visits could not be arranged. There were 15 participants (6 males and 9 females) that completed the experiment. Their average age, height, and weight were 59.6 ± 8.3 years, 160.9 ± 8.3 cm, and 60.3 ± 11.2 kg, respectively. They were all independent walkers. The exclusion criteria included persistent knee pain, osteoarthritis, unstable ankle, severe flat foot, severe hallux valgus, toe deformities, and other chronic diseases that might induce risks in the experiment. The participants also had no history of injuries in the past six months. All participants signed the informed consent, and this study was approved by the Human Subjects Ethics Sub-committee of The Hong Kong Polytechnic University (Reference No: HSEARS20190919001).

### 2.2. Experimental Procedures

**Figure 1:**
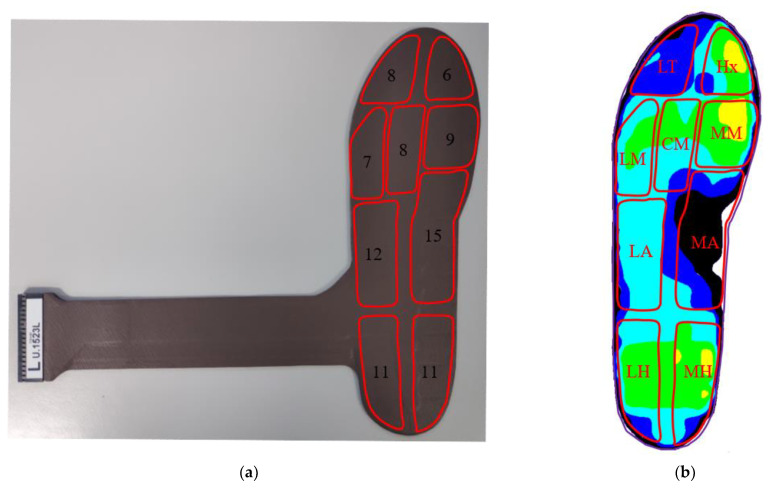
(**a**) The plantar pressure insole used in this study and the number of sensors in each region; (**b**) the plantar pressure distribution of the left foot.

All participants wore the same brand model of running shoes (ARHQ025-4, Li-Ning Inc., Beijing, China) in different shoe sizes, from EU size 36 to 43. The plantar pressure was measured using an in-shoe pressure distribution measuring system, Novel Pedar-X system (Novel Inc., Munich, Germany), which was proven to have excellent reproducibility [39]. The plantar pressure measurement insole (left foot) is shown in Figure 1a, and each insole has 99 capacitive type pressure sensors with a sampling frequency of 100Hz. In addition, the area of single sensor ranges from 151 to 161 mm^2^, with a resolution of 2.5 kPa. The dominant limb was determined by the side of the limb that the participants would use to kick a ball five meters away from the goal [40].

The procedures of the experiment and safety precautions were introduced to the participants before the experiment. The brisk walking task was carried out on a treadmill (Unisen Inc., Tustin, CA, USA). The whole experiment consisted of two parts: the adaptation phase, and a 60-min brisk-walking task. The whole experiment is continuous without pausing.

During the adaptation phase, participants accelerated to their preferred comfortable brisk walking speed (4.1 ± 0.7 km/h) by 0.5 km/h every 30 s [41], then continued to walk at this speed for another 5 min. They achieved the preferred speed in 3.6 ± 0.7 min.

The 60-min brisk walking task was then proceeded at the preferred speed and all participants managed to complete the task. Data collection was carried out during the 60-min brisk walking task only but not for the adaptation phase. Baseline data (1st minute) referred to the first minute of the 60-min brisk walking. After the walking task, participants were asked to indicate verbally whether their level of fatigue was mild, moderate, or severe.

### 2.3. Data Processing

In this study, three time-period data, the baseline (1st minute), the 30th minute, and the 60th minute, were exported. The duration of each time-period is 1 min. As mentioned above, the time here is counted from the beginning of the 60-min walking task. Segmenting the extracted plantar pressure time-series data by stride, a complete stride starts from heel strike to heel strike of the next step in the same side using vertical force and plantar pressure multiplied by area (10 N threshold [42]). The incomplete stride data segment was discarded, and the complete stride data segment was analyzed in this study. The average strides of all time-period data collections are 56.4 ± 3.2.

We divided the plantar foot into nine regions as shown in Figure 1b. The divided regions included hallux (Hx), lesser toes (LT), medial metatarsal (MM), central metatarsals (CM), lateral metatarsals (LM), medial arch (MA), lateral arch (LA), medial heel (MH), and lateral heel (LH) [29,31,32]. The number of sensors contained in the nine regions is as Figure 1a.

**Figure 2:**
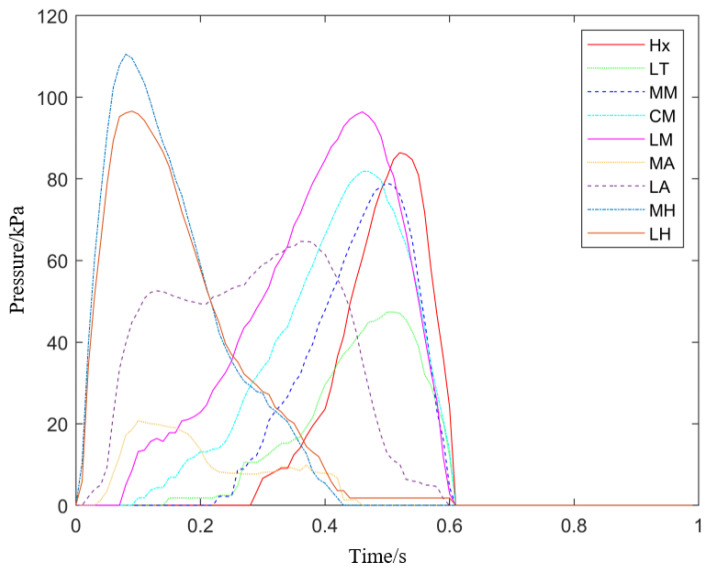
The average plantar pressure of different regions versus time in one gait.

Figure 2 illustrated the regional average plantar pressure versus time in one stride. Contact time, peak pressure, and impulse (pressure-time integrals) in each stride were calculated. The contact time is the projection of the pressure curve above the threshold on the horizontal axis. The pressure thresholds of Hx, LT, MH, and LH are 5 N (1/2 of stride threshold); MM, CM, LM, and MA are 3.33 N (1/3 of stride threshold); LA is 6.67 N (2/3 of stride threshold).

The outcome variables are the median of these variables in all strides in each time condition.

For the calculation of asymmetric index (ASI) of plantar pressure in each region, each piece of stride data is normalized into 101 data time-points (0 to 100% gait cycle) [43]. The average ASI of peak and average plantar pressure are shown in Equations (1) and (2) [13,44]:

(1) $${\mathrm{ASI}}_{pp,reg,t}=\frac{1}{n_{t}}\overset{n_{t}}{\underset{i=1}{\sum }}\frac{\left|pp_{N,t,reg,i}-pp_{D,t,reg,i}\right|}{pp_{N,t,reg,i}+pp_{D,t,reg,i}}\times 100\%,$$

(2) $${\mathrm{ASI}}_{ap,reg,t}=\frac{1}{n_{t}}\overset{n_{t}}{\underset{i=1}{\sum }}\frac{1}{101}\overset{101}{\underset{j=1}{\sum }}\frac{\left|ap_{N,t,reg,i,j}-ap_{D,t,reg,i,j}\right|}{ap_{N,t,reg,i,j}+ap_{D,t,reg,i,j}}\times 100\%,$$

where *ap* is the average plantar pressure; *pp* is the peak plantar pressure; *reg* is the plantar regions; *t* is the three-time conditions: the baseline, the 30th min, and the 60th min; *n_t_* is the strides number in the *t*-th time condition; *i* is the *i*-th stride; *N* and *D* are the non-dominant and dominant feet; and *j* is the *j*-th timepoints (*j*% gait cycle) data.

As a commonly adopted variability index, the median absolute deviation (MAD) is defined as the median of the deviations from the data median [13]. Similar to the average ASI of plantar pressure, each piece of stride data is also normalized into 101 data time-points (0 to 100% gait cycle) for the calculation of MAD of each foot region. Then, the average MADs (all MADs in the corresponding time-series) were obtained for analysis. The data processing procedure was completed by customized program codes (Matlab 2020a, The MathWorks Inc., Natick, MA, USA).

### 2.4. Statistical Analysis

The outcome variables at baseline, the 30th minute, and the 60th minute were compared using the nonparametric Friedman test with a significance level α at 0.05 since some variables did not satisfy the assumption of normality. A post-hoc pairwise comparison using the Wilcoxon signed-rank test with Bonferroni correction at < 0.017 was conducted if significance was found in the Friedman test.

## 3. Results

Eleven participants (73.3%) reported a severe level of fatigue, while there were three (20%) and one participant (6.7%) that reported a moderate level of fatigue and mild fatigue, respectively. Table 1 shows the Friedman test comparing the outcome variables among the three time-periods. In general, the contact time is significantly different in all foot regions; peak pressure in regions except hallux and lateral metatarsal; impulse in regions except hallux and medial metatarsal; ASI of peak plantar pressure in the arch; MAD of average plantar pressure in the arch and medial heel (*p* < 0.05).

### 3.1. Contact Time, Maximum Pressure, and Impulse

A post hoc pairwise comparison with Bonferroni correction was conducted on significant variables according to Table 1, and the results are shown in Table 2.

#### 3.1.1. Contact Time

For the contact time, all regions in both non-dominant and dominant feet showed significant differences in contact time among three-time conditions (*p* < 0.05, Table 1). 

**Figure 3:**
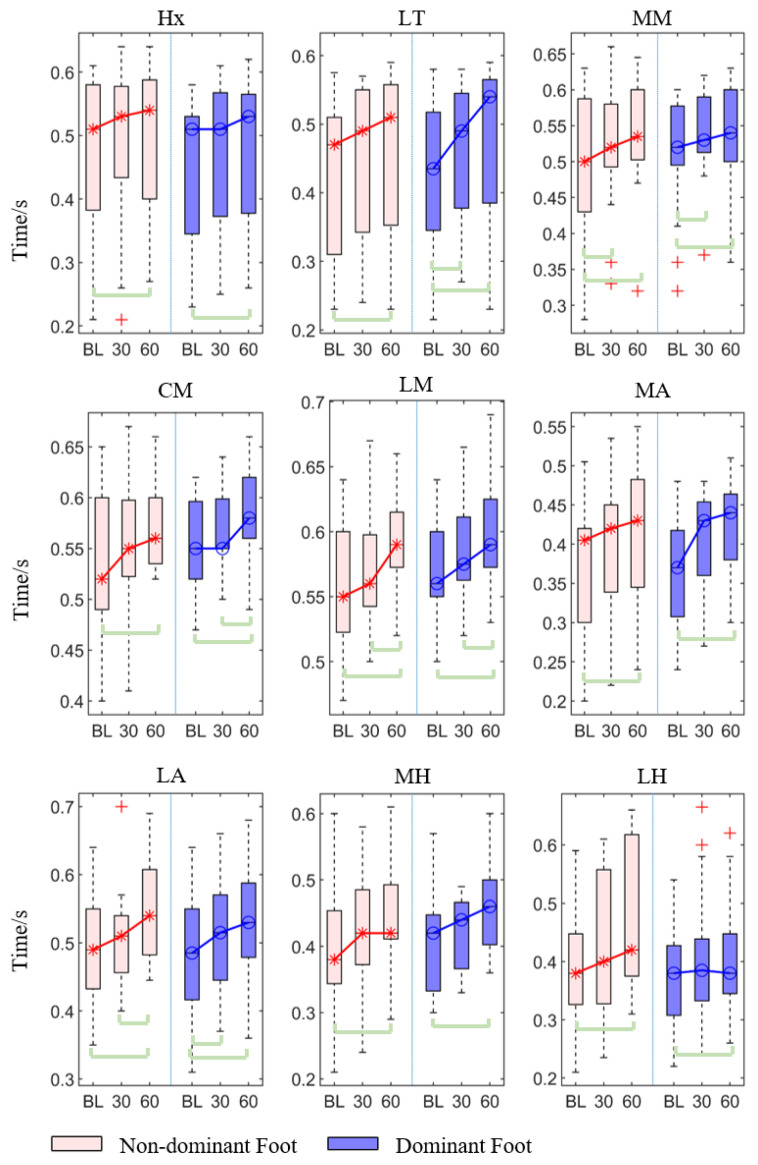
The contact time of each region of the non-dominant and dominant feet at baseline, 30th min, and 60th min. Bracket denotes statistical significance (*p* < 0.05) in the post hoc analysis with Bonferroni correction; BL (baseline), 30 (the 30th min), 60 (the 60th min); Hx (hallux), LT (lesser toes), MM (medial metatarsal), CM (central metatarsals), LM (lateral metatarsals), MA (medial arch), LA (lateral arch), MH (medial heel), and LH (lateral heel).

The post hoc pairwise comparison test (Table 2, Figure 3) showed that there was a significant increase in the 30th min compared with the baseline at the medial metatarsal in both non-dominant and dominant feet (*p* = 0.007, *p* = 0.032), and the lateral toes (*p* = 0.037) and lateral arch (*p* = 0.030) in the dominant foot. Similarly, there was a statistically significant increase in the 60th min compared with the baseline in both non-dominant and dominant feet’s hallux (*p* < 0.001, *p* = 0.030), lateral toes (*p* = 0.023, *p* < 0.001), medial metatarsal heads (*p* < 0.001, *p* < 0.001), center metatarsal heads (*p* < 0.001, *p* < 0.001), lateral metatarsal heads (*p* < 0.001, *p* < 0.001), medial arch (*p* < 0.001, *p* < 0.001), lateral arch (*p* < 0.001, *p* < 0.001), medial heel (*p* < 0.001, *p* = 0.002), and lateral heel (*p* = 0.005, *p* = 0.019). In addition, there was also a significant increase in the 60th min compared with the 30th min in the non-dominant foot’s lateral metatarsal (*p* = 0.026) and arch (*p* = 0.013), and the dominant foot’s center metatarsal (*p* = 0.039) and lateral metatarsal (*p* = 0.013).

#### 3.1.2. Peak Pressure

For the peak pressure, the forefoot (MM and CM) and arch (MA and LA) of two feet showed significant differences among the three-time conditions (*p* < 0.05, Table 1). Similarly, the lateral toes and hindfoot (MH and LH) of the dominant foot also have significant differences among these three-time conditions.

**Figure 4:**
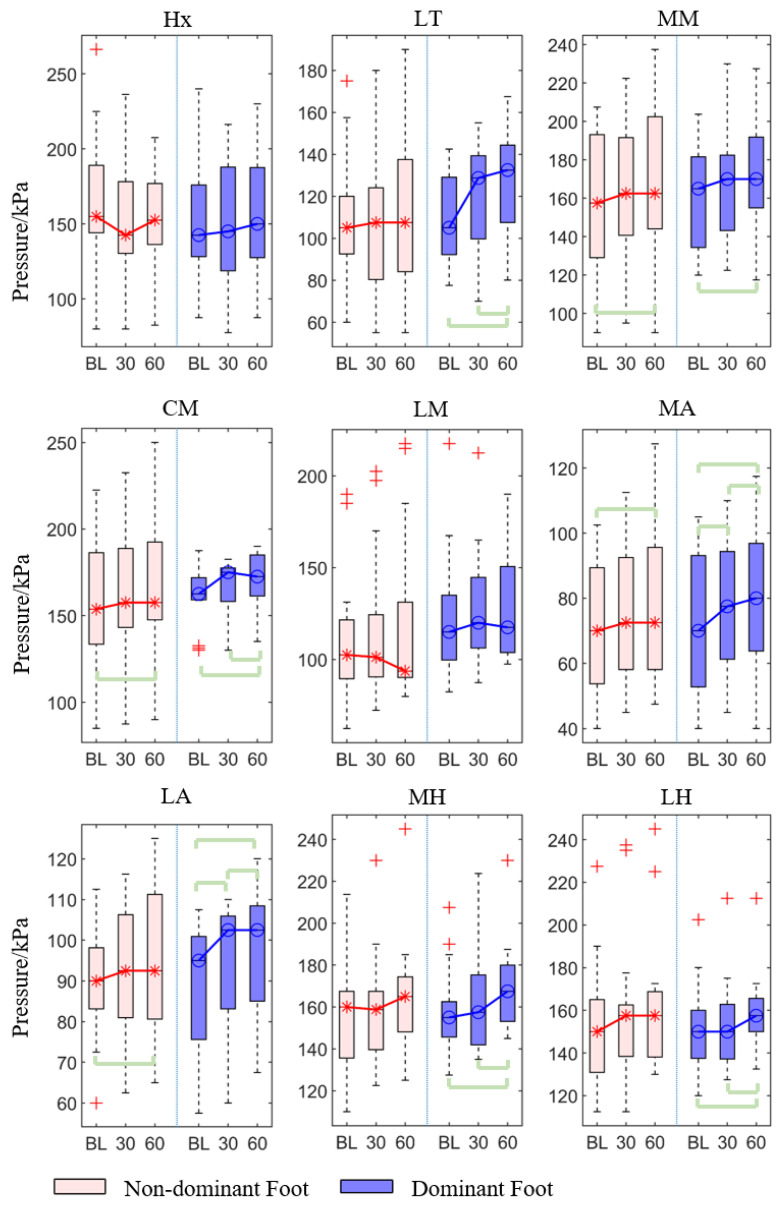
The peak pressure of each region of the non-dominant and dominant feet at at baseline, 30th min, and 60th min. Bracket denotes statistical significance (*p* < 0.05) in the post hoc analysis with Bonferroni correction; BL (baseline), 30 (the 30th min), 60 (the 60th min); Hx (hallux), LT (lesser toes), MM (medial metatarsal), CM (central metatarsals), LM (lateral metatarsals), MA (medial arch), LA (lateral arch), MH (medial heel), and LH (lateral heel).

The post hoc pairwise comparison test (Table 2, Figure 4) revealed that there was a statistically significant increase in 30th minute compared with the baseline in the medial and lateral arch (*p* = 0.016 and *p* = 0.043), for the dominant foot. Similarly, there was a significant increase in the 60th min compared with the baseline in both non-dominant and dominant feet’s medial metatarsal (*p* = 0.002 and *p* = 0.028), center metatarsal (*p* = 0.001, *p* < 0.001), medial arch (*p* = 0.001, *p* < 0.001) and lateral arch (*p* = 0.011, *p* < 0.001), and in the dominant foot’s lateral toes (*p* < 0.001) and medial and lateral heel (*p* = 0.002 and *p* = 0.003). There was also a significant increase in 60th min compared with the 30th min in the dominant foot’s lateral toes (*p* = 0.007), center metatarsal (*p* = 0.024), lateral arch (*p* = 0.043), medial heel (*p* = 0.004), and lateral heel (*p* = 0.035).

#### 3.1.3. Impulse

In this study, pressure time-integral is treated as the impulse. For the impulse, the central metatarsal, arch (MA and LA), and heel (MH and LH) in both feet, and lateral toes and lateral metatarsal in the dominant foot demonstrated significant statistically significant differences among the three-time conditions (*p* < 0.05, Table 1). 

**Figure 5:**
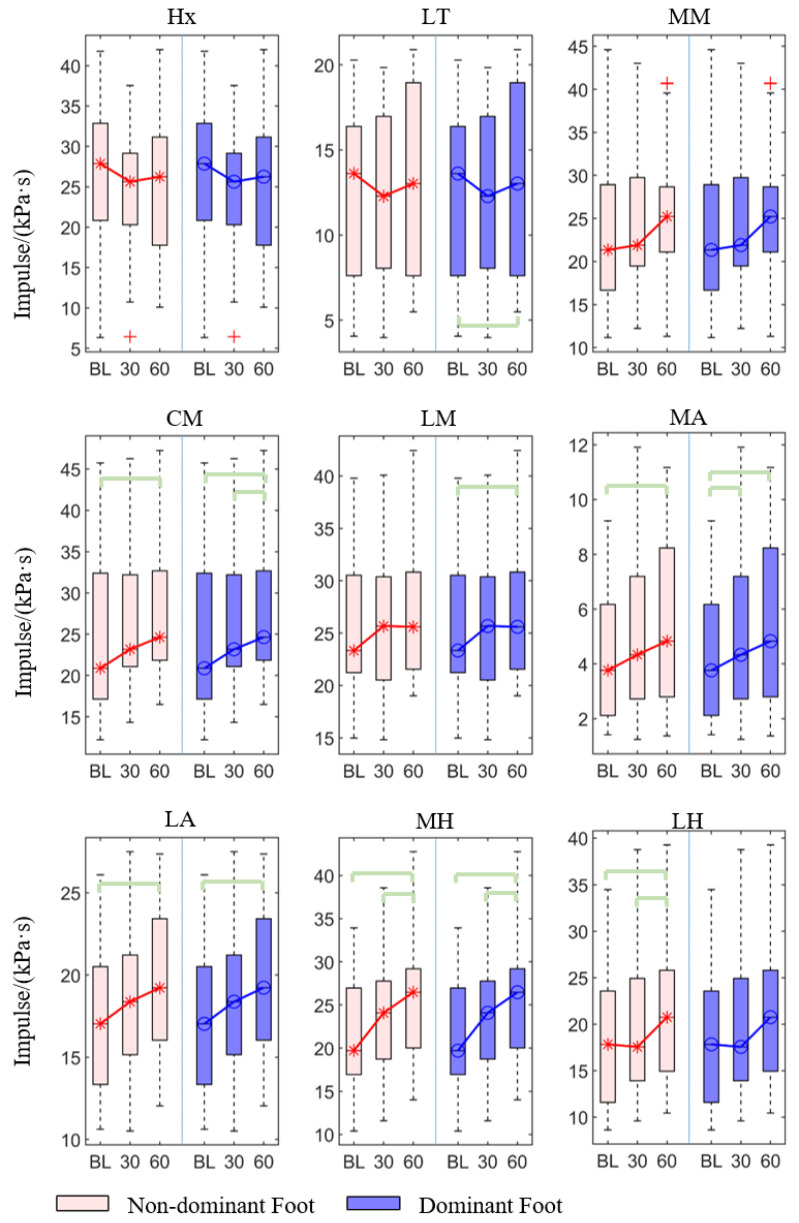
The impulse of each region of the non-dominant and dominant feet at at baseline, 30th min, and 60th min. Bracket denotes statistical significance (*p* < 0.05) in the post hoc analysis with Bonferroni correction; BL (baseline), 30 (the 30th min), 60 (the 60th min); Hx (hallux), LT (lesser toes), MM (medial metatarsal), CM (central metatarsals), LM (lateral metatarsals), MA (medial arch), LA (lateral arch), MH (medial heel), and LH (lateral heel).

The post hoc pairwise comparison test (Table 2, Figure 5) revealed that there was a statistically significant increase in the 30th min compared with the baseline in the dominant foot in the medial arch (*p* = 0.019). Similarly, there was a significant increase in the 60th min compared with the baseline in both non-dominant and dominant feet’s center metatarsal (*p* = 0.019, *p* < 0.001), medial arch (*p* < 0.001, *p* < 0.001), lateral arch (*p* = 0.001, *p* = 0.001), and medial heel (*p* < 0.001, *p* < 0.001), along with in the non-dominant foot’ lateral heel (*p* = 0.010), and dominant foot’s lateral toes (*p* < 0.001) and lateral metatarsal (*p* = 0.002). In addition, there was also a significant increase in the 60th min compared with the 30th min in the medial and lateral heel in the non-dominant foot (*p* = 0.010, *p* = 0.032), and lateral metatarsal heads (*p* = 0.003) and medial heel (*p* = 0.010) in the dominant foot.

### 3.2. Asymmetry Index and Variability

A post hoc pairwise comparison of asymmetry and variability using Bonferroni correction was conducted on significant variables according to Table 1, and the results are shown in Table 3.

#### 3.2.1. Asymmetry Index

**Figure 6:**
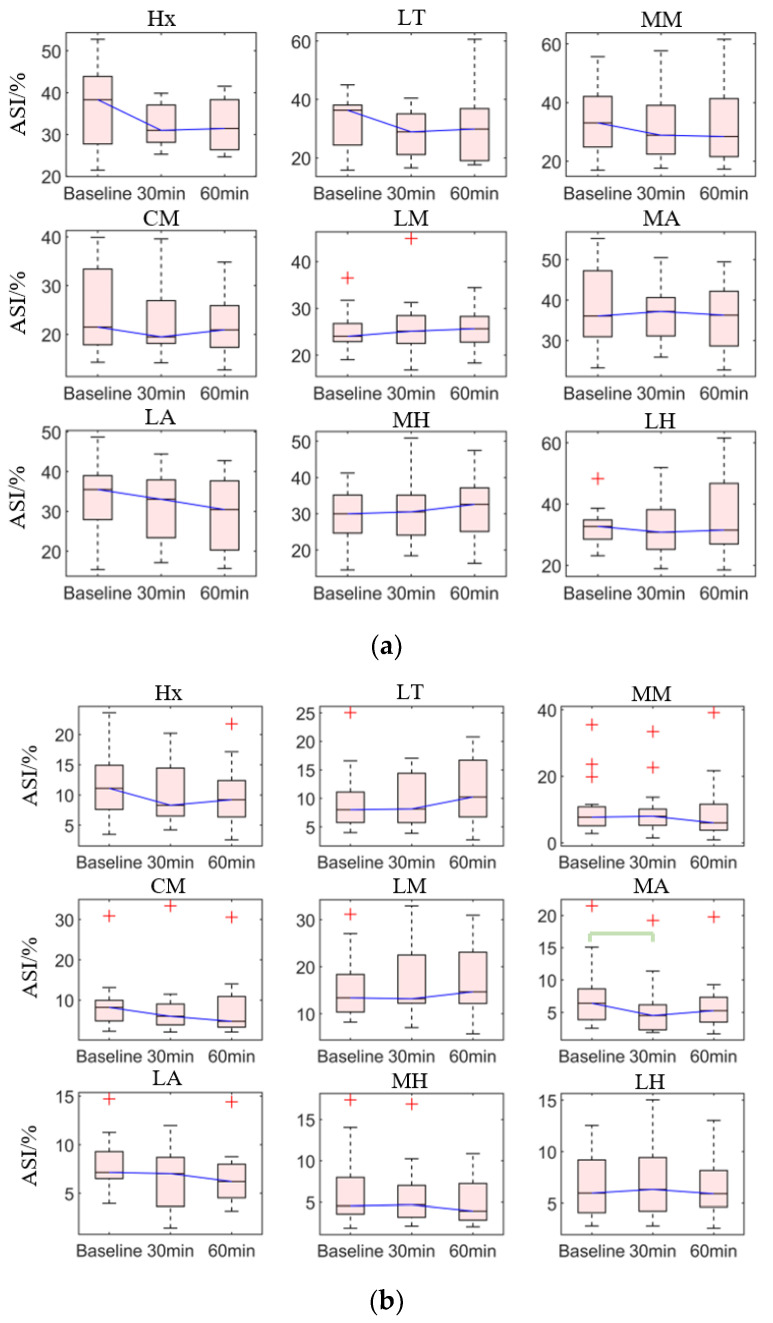
The Asymmetry Index (ASI) of the plantar pressure of each region at baseline, 30th min, and 60th min: (**a**) average plantar pressure; (**b**) peak plantar pressure. Bracket denotes statistical significance (*p* < 0.05) in the post hoc analysis with Bonferroni correction; Hx (hallux), LT (lesser toes), MM (medial metatarsal), CM (central metatarsals), LM (lateral metatarsals), MA (medial arch), LA (lateral arch), MH (medial heel), and LH (lateral heel).

For the ASI of peak plantar pressure between the non-dominant and dominant feet, the medial arch (*p* = 0.008), and the lateral arch (*p* < 0.038) showed significant differences among three-time conditions (Table 1). The post hoc pairwise comparison test (Table 3, Figure 6b) revealed that there was a significant decrease in the 30th minute compared with the baseline in the medial arch (*p* = 0.006), along with a non-significant decrease in the lateral arch (*p* = 0.053). Similarly, there was a non-significant decrease in the 60th min compared with the baseline in the lateral arch. 

For the ASI of average plantar pressure, no pairwise tests were performed since the result of the Friedman test did not show a significant difference. However, data distribution showed that there was a decrease in the 30th minute and 60th minute compared with the baseline in the hallux, lateral toes, medial and center metatarsal, and arch (Figure 6a).

#### 3.2.2. Variability

**Figure 7:**
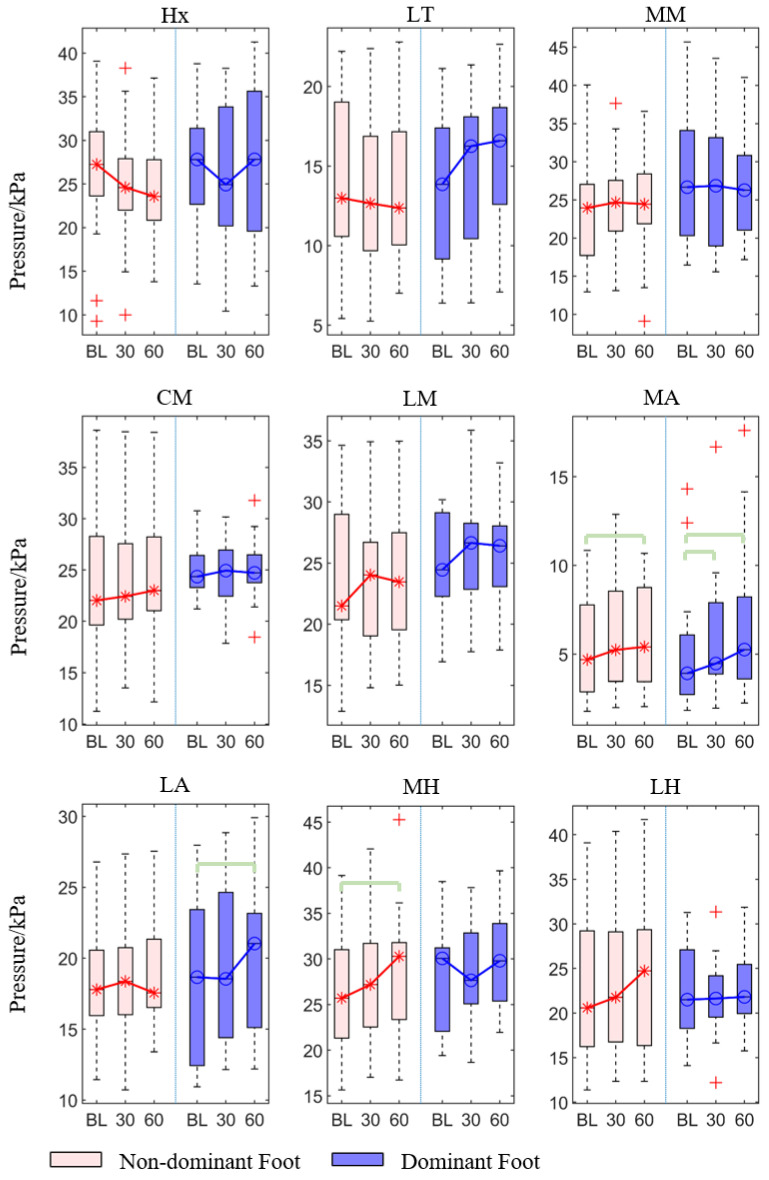
The average median absolute deviation of pressure in each region of the non-dominant and dominant feet at baseline, 30th min, and 60th min. Bracket denotes statistical significance (*p* < 0.05) in the post hoc analysis with Bonferroni correction; BL (baseline), 30 (the 30th min), 60 (the 60th min); Hx (hallux), LT (lesser toes), MM (medial metatarsal), CM (central metatarsals), LM (lateral metatarsals), MA (medial arch), LA (lateral arch), MH (medial heel), and LH (lateral heel).

For the average of MAD, medial arch (*p* = 0.005, *p* < 0.001), lateral arch (*p* = 0.038, *p* = 0.015), and medial heel (*p* = 0.005, *p* = 0.038) in both non-dominant and dominant feet showed significant differences among three-time conditions (Table 1). The post hoc pairwise comparison test (Table 3, Figure 7) revealed that there was a significant increase in the 30th minute compared with the baseline in the medial arch (*p* = 0.019) in the dominant foot. Similarly, there was a significant increase in the 60th min compared with the baseline in the medial arch (*p* = 0.003, *p* < 0.001) in both non-dominant and dominant feet, in the non-dominant foot’s medial heel (*p* = 0.006), and in the dominant foot’s lateral arch (*p* = 0.019).

## 4. Discussion

This study explored the changes of plantar pressure and its asymmetry and variability in elderly adults with fatigue induced by brisk walking. The findings of this study provided preliminary evidence and fundamental confidence for us to develop a fatigue-related instability assessment using plantar pressure sensors in our next step. In this study, after the brisk walking trial, the participants experienced higher peak pressure at the medial and center metatarsal, and midfoot regions, accompanied by an elevated impulse at the center metatarsal, midfoot, and medial rearfoot. In addition, the plantar pressure of the medial arch showed higher variability after the brisk walking trial. Interestingly, the participants attempted to improve symmetry at the medial side of the foot, in addition to an overall increase of contact time. In short, this study demonstrated the impact of lower-limb muscle fatigue on plantar pressure distribution, asymmetry, and variability. 

Neuromuscular fatigue could impair muscular control on the ability to attenuate shock at heel strike [45], resulting in an increase of peak pressure and impulse at the heel region. Compared to the baseline, peak pressure and impulse at heel increase by 2.90% and 7.79% at the 30th min; and 6.05% and 17.43% at the 60th min. The increase of peak pressure and impulse at the heel was aligned with [46], while different to another walking test [32], in which the medial heel showed significantly decreased peak pressure. The difference could be attributed to the fatigue-inducing methods, in which running was adopted in the existing study. 

Reduced supination by the fatigued triceps surae contributed to the increased load at the arch and center metatarsal region [29]. During the midstance phase, compared to the baseline, peak pressure and impulse increased by 6.16% and 20.14% respectively at the 30th min, and 9.96% and 29.85% respectively at the 60th min. At the end of the midstance, peak pressure and impulse at the center metatarsal region increased by 3.53% and 6.71% respectively at the 30th min, and 6.71% and 11.85% respectively at the 60th min. The peak pressure at the medial metatarsal also showed an increase by 3.19% and 6.71% at the 30th min and the 60th min, respectively. These results may explain the reason why metatarsalgia happens after long walking. The impulse at lateral toes in the dominant foot showed a significant increase, which hypothesized that decreased function of the great toe and aims to relieve the pain in the medial and center metatarsal heads affected the rollover process. However, other studies demonstrated mixed findings in the pressure of medial metatarsals and toes, which could be due to the different experimental protocols and characteristics of participants [29,32]. 

The variability of the plantar pressure along the arch and medial heel increased by 13.68% and 4.67% respectively at the 30th min, and 17.92% and 9.20% respectively at the 60th min compared to the baseline. Similarly, center and lateral metatarsal, and lateral heel also increased variability, though not significantly. These results demonstrated a deterioration in stability control [17,20]. The phenomena were supported by other literature [13,26]. In addition, the diminished muscular control over the foot-shank segments may induce external tibial rotation [32], pronounced foot pronation [31], and disturb the normal center of pressure (COP) trajectory [46], which could be the reason for the elevated peak pressure and impulse at the midfoot in this study and that of the previous studies [16,29]. Interestingly, participants showed improvement of peak plantar pressure symmetry at the medial forefoot, medial midfoot, and medial rearfoot, especially at the medial arch by 15.34% and 6.60% respectively at the 30th min and the 60th min, compared to the baseline. They also demonstrated improvement of average plantar pressure symmetry at the medial forefoot and midfoot, though not significant. The improvement could be regarded as a compensatory mechanism in an attempt to restore balance upon further fatigue [13,47]. The compensatory mechanism was facilitated by the prolonged contact time with an increase of 2.73% and 5.82% respectively at the 30th min and 60th min at the whole foot, especially with an increase of 6.50% and 15.32% at 30th min and 60th min at the medial arch. Existing studies suggested that prolonging contact time is beneficial to the adjustment of micro-balance to improve gait stability [9]. For the increased contact time findings, it was consistent with that of existing studies [29,46,48], different from [16,32], possibly due to the different protocol was adopted in this study.

Some studies targeted the joint kinematics and variability measures to assess the fatigue response of elderly during and after exercises. Elhadi et al. [21] and Yeung et al. [49] suggested that the ankle power of the dominant limb was significantly reduced after prolong walking since the plantar flexors were more vulnerable to fatigue. Wong et al. discovered that older adults have a higher variability of joint angular velocity of the ankle joints for both limbs; and that of the knee joint for the non-dominant limb after long-distance walking among older individuals [13]. In another study conducted by Zhang, they mounted an IMU over the heels of elderly subjects [26]. After 60 min of brisk walking, the elderly participants demonstrated significant differences in heel pronation angle, range of angular motion, and variability of angular velocity. On the other hand, Paterson et al. linked the variability of spatiotemporal gait parameters, including step width and step stance, to the falling risks in older adults [50]. Asymmetry and Instability induced by fatigue was also related to poor motor control and thus a predictor of fall and imbalance [51], whereas some research made use of a local dynamic stability methods, such as the maximum Lyapunov exponents, to predict global gait stability during walking [52]. However, it shall be noted that the changing trend of asymmetry and instability during fatigue is not linear, and a sudden restoration may appear due to a compensatory mechanism in an attempt to restore balance [13,47].

Some limitations existed in this study. First of all, “fatigue” is a very broad and abstract concept and can be categorized into neuromuscular, mental, and metabolic [53], and there is no non-invasive method to precise evaluate objectively fatigue so far. In this study, the overall “fatigue” was induced by the long-time brisk walking [26] since most participants reported fatigue after this walking task. In addition, this study heavily relied on the presupposition of the indirect relationship between fatigue and plantar pressure distribution (parameter changes) supported by some literature [16,29,31,32,48]. Physical fitness level, age, sports habits, and footwear could be confounding factors that affected endurance and time-to-fatigue and may induce variabilities along with the time series. This study predicts that participants with similar physiological data could be recruited through inclusion and exclusion criteria. We did not consider the effects of gender, since literature suggested that gender was not associated with the kinematics and kinetics of walking [54]. In addition, treadmill walking may not be the same as overground walking [31], though studies suggested that the kinematic and kinetic gait parameters of these two types of walking are similar [55], while the difference is that participants can reduce the forward propulsion force and still maintain the current speed. This research is limited to the load in the vertical direction and does not consider the load in the medial-lateral and anterior-posterior directions. This study adopted a convenient sampling approach, and future study should consider random sampling in addition to a larger sample size to accommodate for co-covariates for better external validity.

While this study has the abovementioned limitations, it is worth noting that this research facilitates understandings of plantar pressure variables, and their asymmetry and variability upon elderly fatigue and may be considered as an assessment index for gait instability for clinical evaluations. In addition, this article illuminates the prospects for the development of smart devices that identify fatigue levels based on pressure sensors, to enable elderly adults to enjoy fitness keeping while reducing the risk of injury due to fatigue. Future studies may consider the differentiation of myoelectric and metabolic fatigue using electromyography and near-infrared spectroscopy [56]. Future studies can also consider using repeatability analysis of plantar pressure to access the stability of gait [57,58]. In addition, future studies may adopt the inertial measurement unit to achieve wearability [26] and a biofeedback system [59] to achieve interactivity.

## 5. Conclusions

This study explored the effects of long brisk walking induced fatigue on the changes in plantar pressure distribution and its asymmetry and variability in elderly adults. The increase in peak pressure and impulse at the medial heel region may indicate an impaired shock attenuation capability, while the elevated variability along the medial arch may represent gait instability. The improved symmetry and prolonged plantar contact time could be a compensatory mechanism to restore stability upon fatigue.

In this study, we endeavored to induce fatigue-related instability to the elderly through brisk-walking which was proven by their feedback. Some of our targeted asymmetry and variability measures were found to change with the fatigue condition. We therefore believed that these measures could help recognize fatigue-related instability. The outcome of this study can facilitate the development of gait instability or fatigue assessment using wearable in-shoe pressure sensors.

## Figures and Tables

**Table 1:** The Friedman test comparing the three-time periods for the outcome variables, ASI, and MAD.

Variables	Hx	LT	MM	CM	LM	MA	LA	MH	LH
CT_N_	0.001 *	0.021 *	<0.001 *	<0.001 *	<0.001 *	<0.001 *	<0.001 *	<0.001 *	0.007 *
CT_D_	0.023 *	<0.001 *	<0.001 *	<0.001 *	<0.001 *	<0.001 *	<0.001 *	0.002 *	0.022 *
PP_N_	0.437	0.492	0.003 *	0.001 *	0.252	0.001 *	0.015 *	0.061	0.148
PP_D_	0.402	<0.001 *	0.030 *	<0.001 *	0.074	<0.001 *	<0.001 *	0.001 *	0.003 *
IM_N_	0.42	0.247	0.344	0.015 *	0.155	0.001 *	0.001 *	<0.001 *	0.006 *
IM_D_	0.074	<0.001 *	0.127	<0.001 *	0.002 *	<0.001 *	0.001 *	<0.001 *	0.038 *
ASI_ap_	0.074	0.549	0.549	0.420	0.420	0.420	0.627	0.449	0.247
ASI_pp_	0.282	0.344	0.282	0.074	0.282	0.008 *	0.038 *	0.766	0.247
MAD_avg,N_	0.127	0.344	0.819	0.42	0.449	0.005 *	0.038 *	0.005 *	0.057
MAD_avg,D_	0.819	0.189	0.189	0.936	0.091	<0.001 *	0.015 *	0.038 *	0.155

* Statistical difference (*p* < 0.05) using Friedman test; CT_N_ (contact time of non-dominant foot), CT_D_ (contact time of dominant foot), PP_N_ (peak pressure in the non-dominant foot), PP_D_ (peak pressure in the dominant foot), IM_N_ (impulse in the non-dominant foot), IM_D_ (impulse in the dominant foot), ASI_ap_ (the ASI of average plantar pressure), ASI_ap_ (the ASI of peak plantar pressure), MAD_avg,N_ (the average MAD of plantar pressure in the non-dominant foot), MAD_avg,D_ (the average MAD of plantar pressure in the dominant foot), Hx (hallux), LT (lesser toes), MM (medial metatarsal), CM (central metatarsals), LM (lateral metatarsals), MA (medial arch), LA (lateral arch), MH (medial heel), and LH (lateral heel).

**Table 2:** The pairwise comparisons of contact time, peak pressure, and impulse.

Variables	Regions	Baseline-30th min		Baseline-60th min		30–60th min	
		*p*	*p* (Adjusted)	*p*	*p* (Adjusted)	*p*	*p* (Adjusted)
CT_N_	Hx	0.075	0.225	<0.001	<0.001 *	0.039	0.118
	LT	0.519	1.000	0.008	0.023 *	0.043	0.129
	MM	0.002	0.007 *	<0.001	<0.001 *	0.098	0.293
	CM	0.033	0.098	<0.001	<0.001 *	0.016	0.047 *
	LM	0.223	0.670	<0.001	<0.001 *	0.009	0.026 *
	MA	0.028	0.085	<0.001	<0.001 *	0.028	0.085
	LA	0.043	0.129	<0.001	<0.001 *	0.004	0.013 *
	MH	0.053	0.160	<0.001	<0.001 *	0.027	0.081
	LH	0.118	0.353	0.002	0.005 *	0.118	0.353
CT_D_	Hx	0.645	1.000	0.010	0.030 *	0.034	0.103
	LT	0.012	0.037 *	<0.001	<0.001 *	0.164	0.491
	MM	0.011	0.032 *	<0.001	<0.001 *	0.144	0.432
	CM	0.053	0.160	<0.001	<0.001 *	0.013	0.039 *
	LM	0.080	0.241	<0.001	<0.001 *	0.004	0.013 *
	MA	0.028	0.085	<0.001	<0.001 *	0.028	0.085
	LA	0.010	0.030 *	<0.001	<0.001 *	0.021	0.064
	MH	0.144	0.432	0.001	0.002 *	0.045	0.134
	LH	0.273	0.820	0.006	0.019 *	0.100	0.301
PP_N_	MM	0.162	0.485	0.001	0.002 *	0.046	0.137
	CM	0.089	0.267	<0.001	0.001 *	0.047	0.142
	MA	0.032	0.096	<0.001	0.001 *	0.126	0.377
	LA	0.190	0.569	0.004	0.011 *	0.111	0.334
PP_D_	LT	0.095	0.284	<0.001	<0.001 *	0.002	0.007 *
	MM	0.083	0.250	0.009	0.028 *	0.386	1.000
	CM	0.239	0.718	<0.001	<0.001 *	0.008	0.024 *
	MA	0.005	0.016 *	<0.001	<0.001 *	0.054	0.163
	LA	0.014	0.043 *	<0.001	<0.001 *	0.014	0.043 *
	MH	0.773	1.000	0.001	0.002 *	0.001	0.004 *
	LH	0.437	1.000	0.001	0.003 *	0.012	0.035 *
IM_N_	CM	0.584	1.000	0.006	0.019 *	0.028	0.085
	MA	0.100	0.301	<0.001	<0.001 *	0.028	0.085
	LA	0.068	0.204	<0.001	0.001 *	0.068	0.204
	MH	0.201	0.604	<0.001	<0.001 *	0.003	0.010 *
	LH	0.715	1.000	0.003	0.010 *	0.011	0.032 *
IM_D_	LT	0.028	0.085	<0.001	<0.001 *	0.028	0.085
	CM	0.584	1.000	<0.001	<0.001 *	0.001	0.003 *
	LM	0.045	0.134	0.001	0.002 *	0.144	0.432
	MA	0.006	0.019 *	<0.001	<0.001 *	0.028	0.085
	LA	0.018	0.053	<0.001	0.001 *	0.201	0.604
	MH	0.201	0.604	<0.001	<0.001 *	0.003	0.010 *
	LH	0.715	1.000	0.045	0.134	0.018	0.053

*p* (adjusted) is *p*-value adjusted using Bonferroni adjustment; * all pairwise comparison using Wilcoxon signed-rank test demonstrated statistical difference (*p* < 0.05); CT_N_ (contact time of non-dominant foot), CT_D_ (contact time of dominant foot), PP_N_ (peak pressure in non-dominant foot), PP_D_ (peak pressure in dominant foot), IM_N_ (impulse in non-dominant foot), IM_D_ (impulse in dominant foot); Hx (hallux), LT (lesser toes), MM (medial metatarsal), CM (central metatarsals), LM (lateral metatarsals), MA (medial arch), LA (lateral arch), MH (medial heel), and LH (lateral heel).

**Table 3:** The results of ASI and variability.

Variables	Regions	Baseline-30th min		Baseline-60th min		30–60th min	
		*p*	*p* (Adjusted)	*p*	*p* (Adjusted)	*p*	*p* (Adjusted)
ASI_pp_	MA	0.002	0.006 *	0.201	0.604	0.068	0.204
	LA	0.018	0.053	0.045	0.134	0.715	1.000
MAD_avg,N_	MA	0.100	0.301	0.001	0.003 *	0.100	0.301
	LA	0.045	0.134	0.018	0.053	0.715	1.000
	MH	0.018	0.053	0.002	0.006 *	0.465	1.000
MAD_avg,D_	MA	0.006	0.019 *	<0.001	<0.001 *	0.100	0.301
	LA	0.028	0.085	0.006	0.019 *	0.584	1.000
	MH	0.715	1.000	0.018	0.053	0.045	0.134

*p* (adjusted) is *p*-value adjusted using Bonferroni adjustment; * all pairwise comparison using Wilcoxon signed-rank test demonstrated statistical difference (*p* < 0.05); ASI_pp_ (the ASI of peak plantar pressure), MAD_avg,N_ (the average MAD of plantar pressure in the non-dominant foot), MAD_avg,D_ (the average MAD of plantar pressure in the dominant foot), Hx (hallux), LT (lesser toes), MM (medial metatarsal), CM (central metatarsals), LM (lateral metatarsals), MA (medial arch), LA (lateral arch), MH (medial heel), and LH (lateral heel).

## Data Availability

The data presented in this study are available on request from the corresponding author. The data are not publicly available due to privacy issues of the participants.
